# Construction of a Musculoskeletal Discomfort Scale for the Lower Limbs of Workers: An Analysis Using the Multigroup Item Response Theory

**DOI:** 10.3390/ijerph20075307

**Published:** 2023-03-29

**Authors:** Joel Gomes da Silva, Jonhatan Magno Norte da Silva, Lucas Gomes Miranda Bispo, Deividson Sá Fernandes de Souza, Rômulo Silva Serafim, Manoel Gerônimo Lino Torres, Wilza Karla dos Santos Leite, Elamara Marama de Araujo Vieira

**Affiliations:** 1 Production Engineering Course, Backland Campus, Federal University of Alagoas, Delmiro Gouveia 57480-000, AL, Brazil; 2 Department of Production and Transport Engineering, Federal University of Rio Grande do Sul, Porto Alegre 90010-190, RS, Brazil; 3 Department of Production Engineering, Federal University of Pernambuco, Caruaru 55040-900, PE, Brazil; 4 Physiotherapy Course, Federal University of Amapá, Macapá 68903-419, AP, Brazil; 5 Department of Physiotherapy, Federal University of Paraíba, João Pessoa 58051-900, PB, Brazil

**Keywords:** pain symptoms, discomfort metric, multiple professions, lower limb

## Abstract

Musculoskeletal symptoms are a major occupational health problem in workers, and these can affect all professional occupations. Previous studies have proposed metrics capable of evaluating the musculoskeletal discomfort experienced by workers. However, no study has developed a metric that considers professional groups. Thus, this study aimed to develop a scale for musculoskeletal discomfort in the lower limbs to compare self-reported symptoms among education, health, and industry professionals. The sample included 159 teachers, 167 health professionals, and 401 industrial operators who relayed their symptoms using a diagram of the hips, thighs, knees, lower legs, and feet. Factor and multigroup item response theory analyses were used to construct a musculoskeletal discomfort scale consisting of seven levels and to assess and compare the identified symptoms. The results showed that the progressive evolution of discomfort differed for each profession, demonstrating that each context and work environment affects workers differently, which may explain the different patterns of symptom responses among professional groups.

## 1. Introduction

Musculoskeletal disorders (MSDs) are occupational injuries caused or aggravated by work conditions, affecting the joints, cartilage, muscles, nerves, and tendons, among other organs [1,2]. MSDs are considered one of the main occupational health problems in developing countries [3], as they affect all types of professional occupations, including industrial [4], health [5], and education [6] professions. Studies on MSDs have indicated that the upper limbs and lower back region are the most frequently affected regions. However, recent studies have reported prevalence in the lower limbs [2,4,7,8,9], hips, thighs, lower legs, and feet. Regardless of the body region, discomfort is one of the major initial symptoms of any MSD [1].

Discomfort is a subjective phenomenon with physical and mental aspects [10]. Therefore, it is considered a difficult construct to evaluate [11]. Certain instruments, such as questionnaires and diagrams, are used to indirectly evaluate symptoms of discomfort [12]. In addition, metrics are used to verify the level of discomfort in different contexts, such as aircraft seats [10], school desks [13], and musculoskeletal discomfort [14].

Regarding musculoskeletal discomfort, previous studies used the classic test theory (CTT), in which scores are generated through the sum of the responses given to the set of items or body regions [15], as a basis for calculating discomfort scores. Nonetheless, this method suggests that pain symptoms can be added to other symptoms, leading to large bias. Notably, item response theory (IRT) models are among the most accurate and robust methods for mitigating this problem.

According to Silva et al. [12], the IRT model is ideal for evaluating and building scales capable of measuring latent traits (perceived discomfort) related to pain. This is because the method generates scores that vary in a non-linear way when the pain increases or decreases [15]. The scores are calculated from the response patterns of individuals [16], generating continuous scales [15]. Using cumulative IRT methods, it is possible to estimate how the progressive evolution of symptoms occurs in each region of the body [12]. The majority of previous studies have used the IRT model to construct MSD scales, as all workers were part of the same group [12,14,17]. To the best of our knowledge, no study has used a multigroup IRT approach with professionals from different occupations. Job demands and work context tend to vary among different professions. Therefore, analyzing this sample as homogeneous in relation to musculoskeletal symptoms is likely inadequate. In this sense, the IRT multigroup approach can be applied by allowing metrics from different groups to be considered on the same scale through a comparable equalization process [18].

The premise of this study was that self-reported symptoms differ between groups of professionals. Consequently, the response patterns (set of self-reported symptoms) at MSD levels also tend to differ. No metric capable of measuring discomfort in different professionals was found in the literature to reliably compare their response patterns at different levels of MSD. Therefore, this study aimed to develop an MSD scale for the lower limbs capable of comparing and evaluating the progressive evolution of three different groups of professionals (education, health, and industry professionals). For this purpose, a multigroup IRT model was used.

Similarities and differences exist between the work of the three professional groups considered here. For example, health professionals spend almost the entirety of their working day standing and caring for patients or walking between the environments of health facilities. In this environment, patients are transported using stretchers and wheelchairs. However, there are no auxiliary means available to help lift patients or move them from stretchers to beds, requiring high muscle efforts and likely bad postures. Furthermore, health professionals are not qualified or trained to know how to use ergonomic methods during work. The footwear used is purchased by the workers themselves, and the floors of health facilities are built considering architectural aspects (including materials), often resulting in slippery and hard floors. Thus, health establishments are not idealized by considering the anthropometry of health professionals. In addition, the exposure of health professionals to psychosocial stressors is often not considered.

Similar to health professionals, education professionals also perform their activities in a standing position during classes, leading to bad postures when writing due to the position of the boards and the need to flex the abdomen to answer questions from students who remain seated during class. The working hours of these professionals are long, and they often work at more than one educational institution. Moreover, these professionals also spend time sitting and correcting schoolwork, reading teaching materials, and using computers. However, it is unlikely that the furniture, environment, and work instruments used by these professionals at home are planned according to ergonomic principles. In addition, the exposure of these health professionals to psychosocial stressors is often not considered.

Similar to the professionals discussed above, workers in the footwear industry also carry out their work activities while standing and remaining at their workstations. In this role, there is tremendous pressure for production, which requires manual or machine work to be performed quickly and repetitively. Thus, there is no possibility of postural alternation. In addition, the work is monofunctional and idealized in a Taylorist fashion. In this industry, workers wear safety boots and personal protective equipment, which can be uncomfortable. Additionally, the floors of the workstations are rubberized, and while the furniture and machinery have height adjustments, the workers are not trained or qualified to use them.

## 2. Methods

The survey employed in this study was conducted across three sectors: education, industry, and health sectors. The study involved three stages: (I) the selection of the study site and sample, (II) the administration of the research instrument for data collection, and (III) statistical analysis of the collected data. All the procedures used in this study were approved by the Brazilian Research Ethics Committee (CAAE 35014720.6.0000.5013).

### 2.1. Site and Sample Selection

The selection of the study site was based on the sectors studied. The selected education and health sectors were located in the countryside of northeast Brazil. The educational institutions included municipal schools (n = 9), state schools (n = 3), and federal universities (n = 1). The health sector included public hospitals (n = 3) and municipal health centers (n = 3). A footwear factory sector located in northeastern Brazil constituted the industry sector, in which the packaging, assembly, and component preparation sectors were selected as study sites.

To be included in the study sample, workers had to have (1) a minimum age of 18 years and (2) the appropriate physical and psychological conditions to carry out their work activities. The exclusion criteria were (3) temporary employment, (4) history of work accidents, MSD symptoms or any health problem in the last 30 days, (5) hypertension or diabetes, and (6) pregnancy. The sample population consisted of public education teachers (n = 159), footwear industry workers (n = 401), and health professionals (n = 167) working as nurses and nursing technicians.

### 2.2. Administration of the Research Instrument

Data were collected through a questionnaire composed of the following sociodemographic items: sex (male or female), age (in years), length of service (in months), and weight and height. The body mass index (BMI) was determined and expressed as kg/m^2^. An adapted version of the Corlett and Bishop Instrument [19] was used to collect responses associated with the lower limb region (hips, thighs, knees, lower legs, and feet). Four response categories captured the frequency of workers’ reported pain in the respective body regions over the previous 7 days (1 = never; 2 = rarely; 3 = often; 4 = daily). The complete research instrument is presented as Appendix A.

### 2.3. Statistical Analysis

Statistical analyses were performed by separating the data into three professional groups: health (G1), education (G2), and industry (G3). Sociodemographic factors and symptoms were assessed using descriptive statistics. The internal consistency and reliability of the data were tested using Cronbach’s alpha (α) and McDonald’s omega (ωt). Values of ωt > α > 0.70 indicated good data reliability and internal consistency [20].

The Kaiser–Meyer–Olkin (KMO) test and Bartlett’s test of sphericity (BST) were used to fit the data to the factor analysis (FA) method. KMO test values >0.70 and a BST *p*-value <0.05 indicated a good fit of the data to the FA [21]. The dimensionality of the items (body parts) that encompassed the lower limbs of the Corlett and Bishop diagram was verified through exploratory factor analysis and parallel analysis. Items with factor loading (F) < 0.3 and commonality (h2) < 0.2 were extracted from the model [21].

The graded response model [22] of the IRT was used to estimate the discrimination parameter (*a_i_*) of the items and response difficulty categories (*b_ik_*). The latent traits utilized were lower limb MSDs in workers (*θ_j_*). Equation (1) represents the model:

(1) $$P_{ik}\left(\theta_{j}\right)=\frac{1}{1+e^{-a_{i}\left(\theta_{j}-b_{ik}\right)}}-\frac{1}{1+e^{-a_{i}\left(\theta_{j}-b_{ik+1}\right)}}$$

where $P_{ik}\left(\theta_{j}\right)$ is the probability that worker j chooses the response category *k* for item *i*, considering *b_i_*_2_ < *b_i_*_3_ < *b_i_*_4_.

The parameters were estimated with a mean of 0.00 and a standard deviation of 1.00. The method of parameter equalization via multiple groups [23] was used such that the IRT parameters were placed in the same metric. The IRT parameters were linearly transformed from scale (0 ± 1) to scale (50 ± 10), as described by Menegon et al. [10].

The instrument’s information curve indicates the region of the scale with greater precision; that is, the region of the scale constitutes the greatest amount of information and the smallest measurement error, which is an important validation indicator of the data collection instrument [24]. Equation (2) shows the calculation of the information for each item [25]:

(2) $$I_{i}\left(\theta \right)=\sum_{X=1}^{ki}\frac{{P´}_{ix}{\left(\theta \right)}^{2}}{{P´}_{ix}\left(\theta \right)}$$

where ${P´}_{ix}\left(\theta \right)$ is the first derivative of the response curve of the category evaluated at a given level of the latent trait; therefore, greater discrimination of item *i* implies that more information is provided to the measurement instrument [12].

The scales were constructed using an anchoring process. Thus, Z anchor levels were defined in the same unit as the latent trait (lower limb MSDs of workers, *θ_j_*). The levels were spaced 10 units of *θ_j_* apart, generating the levels in which we sought to generate the scale’s response patterns. The values of the Z anchor levels, as well as the discrimination (*a_i_*) and difficulty (*b_ik_*) parameters of each item *i*, were replaced in the equation to calculate the conditional and cumulative probabilities of the IRT. A conditional probability value was then calculated for each Z anchor level as a function of a_i_ and each *b_ik_* related to the *k* − 1 response alternatives.

The answer alternative *k* for item *i* was anchored in the first Z level with a cumulative conditional probability >50%. In summary, the scale was generated from the Z anchor levels that presented some of the *k* response categories that were anchored between the different *i* items of the research instrument. Therefore, the set of *k* anchored response alternatives for all *i* items generated the response patterns for each anchor level Z of the constructed lower limb discomfort scale. For each anchor level Z, along with the *k* anchored answer alternatives, qualitative meaning was attributed to the function of the anchored answer alternatives. The scale was built with meanings and response patterns linked to each level. The response patterns of the three professional groups were compared (according to sex, age, length of service, and BMI) at all levels of the discomfort scale using the Kruskal–Wallis test. All statistical procedures were performed using R software version 4.1.2 [26].

## 3. Results

The collected data showed α and ωt values greater than 88% and 92%, respectively, for the three groups. These results indicated satisfactory data reliability (ωt > α > 0.70). In addition, KMO > 0.70 and BST *p* < 0.05 indicated a satisfactory fit to the FA.

### 3.1. Data and Statistical Analysis

Table 1 summarizes the characteristics of the workers in the health (G1), education (G2), and industry (G3) sectors. The workers in the three groups had 6–10 years of experience at their respective companies. The majority of the population in G1 and G2 were women aged 41–50 years. In G3, most participants were men aged 21–30 years. Regarding BMI, G2 and G3 had normal weights, while G1 was overweight.

Table 2 shows the self-reported MSD symptoms of the workers in the three groups. The data show that within each group, there were professionals who reported infrequent, frequent, and daily pain symptoms in all evaluated body groups. However, the highest prevalence of pain was different in each group: in G1, pain in the knees and lower legs was predominant at approximately 35%; in G2, this was 51% in the hips. In G3, pain was predominant in the thighs, with an average of 74%. In addition, industry professionals had a higher prevalence of symptoms than education and health professionals in most of the regions studied.

The table above shows that in G1, the major regions associated with daily pain were the hips and left knee (approximately 13%). Regarding frequent and daily pain, approximately 20% of symptoms were prevalent in the lower legs and feet. In G2, the prevalence of daily symptoms was >18% in the lower legs and hips, and the prevalence of symptoms perceived as frequent and daily was greater than 21% in most regions, except for that in the right thigh. G3 professionals reported daily pain greater than 11% in the lower limbs, except in the lower legs. In the thigh, knee, and foot regions, there was a higher prevalence of frequent and daily pain in G3 professionals than in G1 and G2 professionals.

### 3.2. Dimensionality and Item Parameter Estimation

Table 3 presents the FA and IRT estimates for G1, G2, and G3. The analysis of these parameters verified the quality of all items by expressing F and h2 values > 0.400 and 0.200, respectively, in a single dimension [21]. The proportion of variation linked to this factor was >30% for all groups, indicating that a single dimension explained the latent trait of discomfort in the lower limbs [12,14]. The IRT parameters of discrimination and difficulty (a_i_ > 0.70 and b_i_ ∈ [−3, 3]) were satisfactory and did not require the exclusion of any body region [27,28].

**Figure 1:**
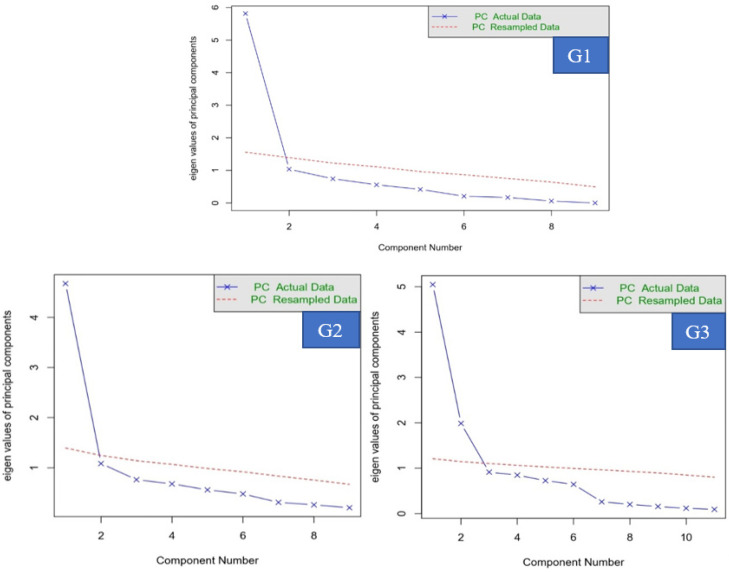
Parallel instrument analysis chart for the three groups.

Figure 1 presents the graphs of the parallel analysis for each group. The FA was verified, wherein one dimension explained the latent trait due to the presence of an eigen value significantly greater than the others. This indicates that the instrument’s items are adequate, confirming that the latent trait is one-dimensional.

### 3.3. Instrument and Information Tests

**Figure 2:**
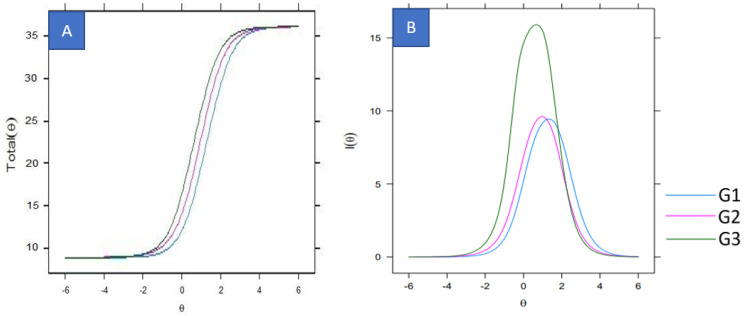
Instrument (**A**) and information curve (**B**) graphs.

The instrument and information curves are presented for the health (G1), education (G2), and industry (G3) workers (Figure 2). The instrument curves were similar for the three groups, indicating that the set of items had a similar ability to discriminate workers with different levels of discomfort from G1, G2, and G3.

When analyzing the information curves, greater symmetry was observed for industry workers (G3), with more information captured around θ = 0.5, with values varying between [−1.0, 2.5]. In contrast, G1 and G2 concentrated more information on θ = 1.0 and θ = 1.5, with values varying between [0.0, 3.0] and [−0.5, 2.5], respectively. In the analysis of the G3 information curve, the instrument items generate better information for industry workers, achieving a value closer to the average discomfort level. Meanwhile, it still captured an adequate amount of information for individuals with a level of discomfort just below the mean (approximately 1 standard deviation) and for those with discomfort levels well above the mean (approximately 2.5 standard deviations). Regarding workers from G1 and G2, the instrument’s items were appropriate for measuring discomfort in the lower limbs of the health and education professionals, revealing a level of discomfort slightly above the average (1–1.5 standard deviations above an average discomfort value). Notably, this is the region with the greatest amount of information for these sample groups. Similarly, the items did not adequately measure the level of discomfort in the lower limbs of the education and health professionals with discomfort levels much lower than the average (θ < 0.5); however, they did provide accurate measures for professionals with a high level of discomfort (θ > 2.5).

### 3.4. Lower Limb Discomfort Scale

Table 4 presents the response alternatives for the scale developed to measure discomfort in the lower body. To generate the scale, the items were anchored at seven different levels, which indicated the workers’ response patterns as a function of their respective body discomfort. Levels 45, 50, 55, 60, 65, 70, and 75 were associated with minimal discomfort (level 1), mild discomfort (level 2), low discomfort (level 3), moderate discomfort (level 4), high discomfort (level 5), severe discomfort (level 6), and maximum discomfort (level 7), respectively. On the scale, the scores of the individuals (answer alternatives) were interpreted at each level: A1 meant they never felt pain (reference value), A2 meant they rarely felt pain, A3 meant they often felt pain, and A4 meant they felt pain daily.

**Figure 3:**
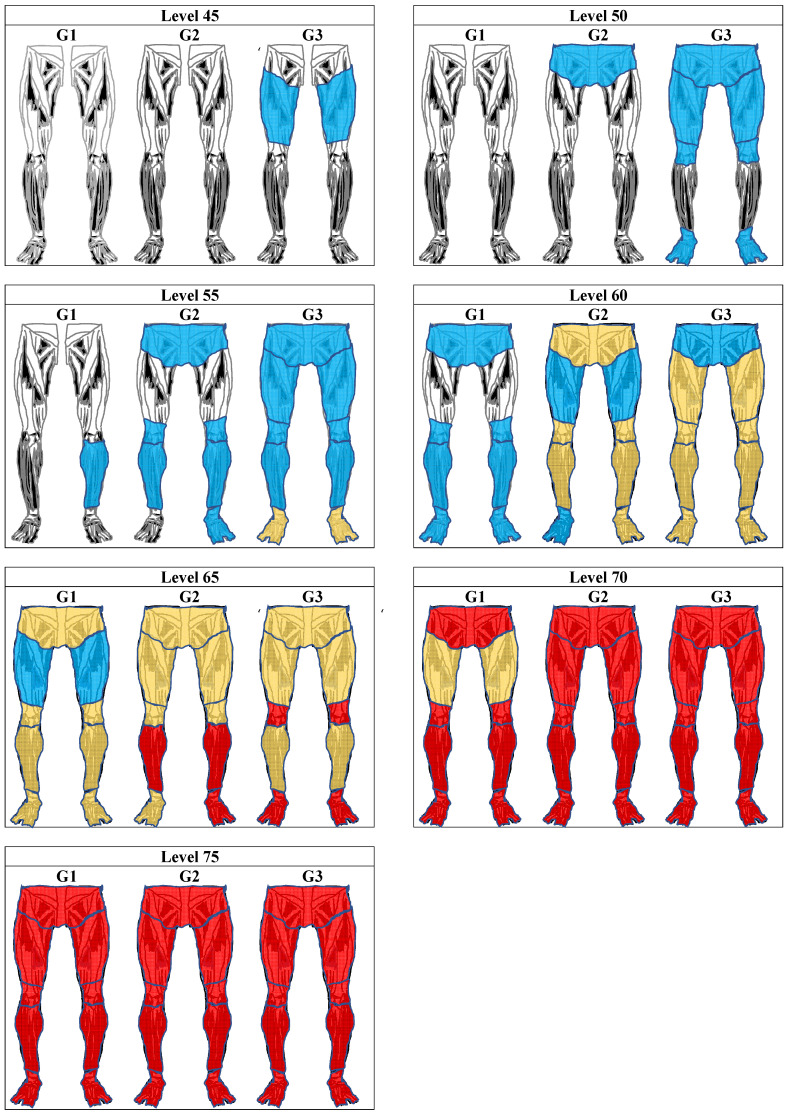
Evolution of lower limb musculoskeletal discomfort for the three groups at each scale level. Legend: blue, rarely feels pain; yellow, often feels pain; red, feels pain daily.

According to the scale above and its metrics, workers in the health (G1), education (G2), and industry (G3) sectors had different response patterns (Figure 3). Thus, even if they had the same level of discomfort in a body region, their answers to the questionnaire items tended to differ. The G3 workers’ response alternatives began to be anchored at level 1, whereas this was level 3 for G2 and from level 4 onward for G1. As the scale was developed from a cumulative model (graduated response model), it is expected that an industrial worker, for example, with minimal discomfort (level 45) will start with symptoms (pain rarely or A2) in the left and right thighs, which were the items anchored at this scale level. As the level of discomfort increases, these workers will then begin to experience frequent or daily pain. Finally, in the three groups, the last level of discomfort represents situations in which the worker feels symptoms in all regions of the lower limbs of the body.

The Kruskal–Wallis test was used to compare the response patterns of the three professional groups according to sex, age, length of service, and BMI at each level of the lower limb discomfort scale (Appendix B). Regarding sex, at level 45 of the scale, some response patterns were significantly different: men from the health sector and men (*p*-value = 0.0452) and women (*p*-value = 0.030) from the footwear industry showed different response patterns. However, values close to statistical significance were observed among women in the health sector and in the footwear industry (*p*-value = 0.0531), men in the education sector and women in the footwear industry (*p*-value = 0.0823), and women in the education sector and in from the footwear industry (*p*-value = 0.0929). At levels 55 and 60, differences were also observed in the response patterns of men in the health sector and women in the footwear industry, with p-values equal to 0.0041 and 0.054, respectively. Furthermore, at level 75, differences between women in the health sector and men (*p*-value = 0.0322) and women in the footwear industry (*p*-value = 0.0030) were found. At the same level, values close to statistical significance were observed among women in the health sector and women in the education sector (*p*-value = 0.0894).

Regarding age, at level 45 of the scale, there was a significant difference in the response patterns among health workers aged less than 30 years and workers in the footwear industry aged up to 49 years (*p*-value = 0.0376) and over 50 years (*p*-value = 0.0056). In addition, differences in response patterns were observed between health workers aged up to 49 years and workers in the footwear industry aged less than 30 years (*p*-value = 0.0452) and up to 49 years of age (*p*-value = 0.0089). This same difference was observed at level 55 of the scale for the same group of workers (*p*-values of 0.0240 and 0.0030, respectively). Furthermore, values close to statistical significance were observed among health workers aged over 50 years and workers in the footwear industry aged up to 49 years (*p*-value = 0.0982) and over 50 years of age (*p*-value = 0.0676). Additionally, this was observed among education workers aged up to 49 years old and in footwear industry workers aged up to 49 years old (*p*-value = 0.0746). At level 60 of the scale, a difference was observed between health workers aged less than 30 years and workers in the footwear industry aged up to 49 years (*p*-value = 0.0298). For health workers aged up to 49 years, this difference continued to exist in relation to workers in the footwear industry (*p*-value = 0.0011). At level 75, differences were observed between health workers under 30 years old and workers in the footwear industry under 30 years old (*p*-value = 0.04977) and up to 49 years old (*p*-value = 0.0305).

Workers’ length of service also led to differences in response patterns. For example, at level 45 of the scale, a difference in the response pattern was observed between education professionals with less than 10 months of service and footwear industry workers with up to 20 months of service (*p*-value = 0.0025). This same difference was observed at levels 55 and 60 of the scale. At level 75 of the scale, there was a difference in the response pattern between health workers with less than 10 months of service and workers in the footwear industry with less than 10 months of service (*p*-value = 0.0248).

Finally, BMI also led to different response patterns across the professional groups. For example, overweight healthcare workers and underweight or normal-weight footwear industry workers showed different response patterns at levels 45, 55, 60, and 75. Furthermore, at level 45 of the scale, education professionals that were underweight or normal weight showed different response pattern than workers in the footwear industry who were underweight or normal weight (*p*-value = 0.0344). In addition, at level 75 of the scale, overweight health professionals showed a response pattern similar to that of workers in the footwear industry with any BMI classification. Thus, the existence of different response patterns at the scale levels among health, education, and footwear industry professionals was reinforced, and aspects such as sex, age, length of service, BMI, and work demands can explain the different groups of symptoms reported by workers.

## 4. Discussion

The indirect quantification of certain variables, such as the perception of MSD, is challenging, as musculoskeletal symptoms cannot always be reliably evaluated using electronic equipment [29]. In ergonomics, the use of the CTT, that is, the sum of responses from survey instruments to generate scores, is the most common method; however, the measurement errors of the CTT are higher than those of the IRT [30]. In terms of pain symptoms, there is a greater error and bias associated with the simple or weighted sum of pain symptoms, with response patterns generated from IRT parameters being the most appropriate and accurate measure to determine individuals’ scores [15]. This is because isolated pain symptoms are much less common than multiple symptoms [31]. Therefore, the set of symptoms present can be captured at each level of MSD [14]. By analyzing each level of the scale generated by the IRT and the respective pain symptoms (which form the response patterns), symptoms can be monitored as they worsen [12].

Researchers such as Hamberg-van Reenen et al. [32] have already theorized that cumulative models, such as the IRT model used in this article, can help predict future symptoms. The results generated using the IRT approach overcome some of the limitations of CTT methods. Nonetheless, their use in ergonomics remains limited [30]. In the case of a multigroup IRT approach, literature on musculoskeletal symptoms is scarce [18]. Thus, this study is the first to propose a scale based on symptoms reported by workers from different professions. Notably, most previous studies using IRT models did not account for the characteristics that could interfere with parameter estimation. Additionally, they did not verify that these differences were absent in the evaluated samples [33]. Therefore, studies on ergonomics have overlooked the differences [10,12,13,14,34].

When using multigroup IRT models, workers from different professions are placed on the same metric, enabling comparisons between their levels of discomfort. This is because the IRT allows for an equalization process in which the item parameters and latent traits of respondents from different groups can be analyzed, understood, equated, and compared once they are on the same metric or common scale [35]. The parameters of the items in the different groups are in the same metric. Nonetheless, they tend to assume different values, allowing the result of the anchoring process to generate different response patterns for each group [18]. Thus, in this study, it was possible to analyze the different response patterns of samples from occupational groups in the health, education, and industry sectors. As the working environment of each profession requires specific work activity demands, a different and progressive illness process was expected between the groups. However, how the aggravation of symptoms in the lower limbs occurs remains unknown.

Based on the analysis of response patterns, symptoms in the lower limbs reported by professionals began to appear at different times according to their occupational group. Industrial workers, for example, begin to experience symptoms (infrequent pain) in their thighs at the lowest level of discomfort on the scale. In contrast, the other occupational groups did not present any symptoms at that level. Furthermore, only those in the industry sector showed progression of symptoms simultaneously on the left and right limbs.

### 4.1. Health Workers

Recent studies have shown high rates of MSDs in health professionals [5], although quality studies focusing on interventions that can prevent musculoskeletal injuries among such workers are limited [36]. As a result, such professionals frequently seek medication to relieve symptoms [37], have problems with insomnia [9], and consider changing professions [38]. MSD symptoms in healthcare workers reduce workers’ professional performance and contribute to an increased burden for the team [39], which affects patient safety.

On the discomfort scale in the lower limbs, symptoms were anchored at four levels ranging between low 60 > θ_j_ ≥ 55) and maximum discomfort (θ_j_ ≥ 75). Thus, in situations of minimal and mild discomfort, health professionals tended not to report any musculoskeletal symptoms in the lower limbs. The reason none of the response alternatives are anchored in the first two levels of the scale remains unknown. However, regions of the body in which health professionals frequently report symptoms, such as the back [40] and lumbar region [41], were not considered in this scale, which may have resulted in the absence of a response pattern at such levels. In this study, we chose to focus on the lower limbs owing to the low number of studies evaluating symptoms in these specific regions of the body in health professionals [5].

At the low discomfort level (60 > θ_j_ ≥ 55), the presence of infrequent symptoms tended to start in the right leg. Notably, symptoms are commonly much more concentrated in the lower part of the body and on the right side in health professionals [9]. This is because these professionals spend a significant part of their workdays standing, which directly contributes to leg symptoms [41]. Corroborating this, Ribeiro et al. [42] highlighted that more than half of health professionals reported symptoms in the lower legs.

At the level of moderate discomfort (65 > θ_j_ ≥ 60), infrequent symptoms began in the left leg, hips, knees, and feet. Therefore, at this level, the number of regions with pain symptoms increased. Regarding health professionals, efforts related to patient transport and the need to adopt inappropriate postures worsens these symptoms [42]. Such biomechanical efforts require the application of forces not only by the upper limbs but also by several other segments of the body. For example, in the studies by Engholm and Holmström [43] and Andersen et al. [44], efforts related to lifting with the hands and pushing resulted in an increase in the risk of knee symptoms. In addition, the need for manual activity has also been observed as a risk factor for foot symptoms in workers [2]. Furthermore, the use of ill-fitting or inappropriate shoes is also associated with foot pain [45]. Thus, as reported in a study by Chiwaridzo et al. [37], prolonged exposure times may explain the greater number of symptoms among more experienced health professionals.

At the level of high MSD (70 > θ_j_ ≥ 65), existing symptoms worsened and became increasingly frequent, and symptoms began to develop in previously symptom-free regions such as the thighs (left and right). Certain studies have pointed out that symptoms in the thighs of health professionals may be more frequent than those in the back and upper limbs [46,47]. However, in our sample, these symptoms were only reported in the presence of more severe symptoms in other lower limb regions. This set of symptoms that affect all regions of the lower limbs can also be explained by the fact that these professionals are required to walk and perform work for long periods on hard and slippery floors [5,47]. These situations lead to an overload that cumulatively affects all lower limb regions. 

At severe discomfort levels (75 > θ_j_ ≥ 70), symptoms occurred daily in the knees, lower legs, feet, and hips and frequently in the thighs. Therefore, worsening of symptoms can be observed, with pain being increasingly present in the daily lives of professionals. Bispo et al. [2] found that in the countryside of the states of Alagoas and Bahia, the design of the workstations was not appropriate. This increased the likelihood of symptoms in the lower limbs owing to the need to maintain the lower limbs in inappropriate postures and the high biomechanical load resulting from moving patients. However, Alhazim et al. [48] highlighted that redesigning workstations is not enough to prevent MSDs, and efforts should also focus on labor practices, especially those related to ergonomics. Thus, inadequate workstations and absence of ergonomic practices are ideal conditions for worsening symptoms in the lower limbs. However, few studies have investigated more severe MSDs in health professionals [5], which hinders our understanding of the factors that worsen MSD symptoms. However, certain data indicate that social support can reduce the damage of high physical demands on the severity of MSDs among health professionals [49].

Finally, at the maximum level of discomfort (θ_j_ ≥ 75), health professionals reported daily symptoms in all regions of the lower limbs, with a particular emphasis on the thighs, which are often the last to develop daily symptoms. The findings by Bispo et al. [2] indicate that, although maintaining the lower limbs in an uncomfortable position is a factor that increases the chance of symptoms in the thighs, psychosocial stressors (such as a low motivation, low support from co-workers, overcommitment, and low job control) greatly influence the symptoms in this region of the body. In addition, there is evidence that psychosocial stressors have an indirect effect on MSDs [50,51], resulting in increased muscle tension and perceived workloads [52,53]. Pain symptoms are also sources of stress, contributing to more severe symptoms [54]. Thus, the scale developed here can help identify the discomfort of health professionals as they are exposed to numerous physical occupational risks [7], including pulling, pushing, lifting instruments and equipment, and moving patients [55], but also to high psychological and psychosocial demands [49].

### 4.2. Education Workers

Education workers exhibit high rates of MSDs [6]. However, research is scarce on MSD patterns and risk factors among teachers in educational institutions [56,57,58]. Studies such as the one performed by Erick and Smith [59] show that MSDs in the lower limbs, including the hips, lower legs, knees, ankles, and/or feet, were reported by 41.1% and 33% of teachers in Brazilian schools and US preschools, respectively. Furthermore, the prevalence of MSDs among schoolteachers are high, ranging between 40% and 95% [60,61].

Education professionals are exposed to high-risk factors, including incorrect postures while writing on the blackboard, head-down postures while reading, and other inappropriate postures such as when bending over while teaching and assisting students with learning difficulties. These professionals are also exposed to long standing hours during teaching, repetitive movements including the use of stairs, physical education activities, and prolonged sedentary positions when planning lessons and recording student results [62]. Thus, they may be a high-risk group for work-related pain in the lower limbs [63].

The MSD scale for the lower limbs anchored symptoms into seven levels, ranging from mild (55 > θ_j_ ≥ 50) to maximum discomfort (θ_j_ ≥ 75). In this group, no response pattern was noted at the minimal discomfort level, indicating that teachers did not usually report pain symptoms when their level of discomfort was very low.

At the level of mild discomfort (55 > θ_j_ ≥ 50), infrequent symptoms were reported in the hip region. Souza et al. [51] observed that teachers in the countryside of Alagoas and Bahia often had two employment contracts, which lead to long weekly workdays. Erick and Smith [59] and Cardoso et al. [60] reported that for professionals in Brazil, working >40 h a week and having >30 students in a class were associated with the onset of pain in the hip region. Nonetheless, >30 students is frequently experienced by professionals in small cities in the countryside. According to Acaröz Candan et al. [64], working up to 4.5 h in a static position with excessive posterior inclination of the pelvic region contributes to pain symptoms in this region. However, time spent in a sitting position also has broad implications for the health of professionals. Several studies have also suggested that repeated activities performed by schoolteachers in a prolonged sitting posture, such as while reading, marking assignments, or using the computer, are the causes of MSDs [56,59,63,65].

At the low discomfort level (60 > θ_j_ ≥ 55), infrequent symptoms beyond the hip region were reported, such as those affecting the right foot, knees, and lower legs. Findings by Vega-Fernández et al. [6] and Lizana et al. [66] indicate that knee pain may be associated with the high prevalence of reported obesity. Moreover, teachers who do not engage in physical activity have more significant obesity rates than those who do [58,67,68]. In a different study, Anderson et al. [45] found that prolonged periods of standing and maintaining an upright posture were associated with an increased risk of MSDs in the lower legs and right foot. Furthermore, according to Alias et al. [58], standing for up to 4 h and poor choices of footwear during school hours contribute to symptoms in the feet and lower leg regions. Moreover, Lima da Silva et al. [69] found that jobs in the countryside of northeastern Brazil are precarious and have low wages. Thus, these professionals are often unable to purchase comfortable shoes, only having access to basic necessities.

At the level of moderate discomfort (65 > θ_j_ ≥ 60), symptoms were usually already present in all regions of the lower limbs. In addition, infrequent symptoms were reported in the left foot and thighs. Additionally, common symptoms were reported in the right foot, knees, and lower legs. According to studies by Alias et al. [58] and Smith [70], pain in the knees and thighs occurs among teachers during working hours due to prolonged stays at schools for several hours and the frequent climbing of stairs to reach the upper floors. According to Leme and Maia [71], the degree of discomfort in the feet may be due to the way employees position themselves to teach, with their body weight held in standing postures for long periods of time. Several studies have highlighted that forced work positions, long standing hours, prolonged static postures, repetitive movements, poor postures, genetic predispositions, stress, poor physical conditions, age, and obesity are risk factors for disorders of the hip and lower leg region [59,64,72,73]. Notably, many of these occupational risk factors were identified in the analyzed region. Indeed, education professionals are subject to such employment conditions owing to the low number of jobs and intense competition in cities in the Brazilian countryside [2].

At high discomfort levels (70 > θ_j_ ≥ 65), symptoms became frequent in the left foot and thighs and daily in the lower legs and right foot. According to Alias et al. [58], teaching for 1–4 h, in addition to engaging in sports activities during school hours, is significantly correlated with the occurrence of disorders in the thighs and feet. Furthermore, limiting pain in these regions is greater for rural teachers than for teachers working in urban regions [57]. This is because teachers in rural areas are more at risk of developing MSDs and face greater challenges, such as social and geographic isolation and poorer working conditions, than those in urban areas. At the same level, in addition to experiencing high levels of discomfort, education professionals reported initial daily pain symptoms in the lower legs and right feet. According to Alias et al. [58] and Vaghela and Parekh [61], exposure to standing hours is significantly related to MSDs in the lower leg and foot regions, increasing the chance of pain among education professionals by 1.75 and 1.02 times, respectively. This is explained by daily teaching activities such as writing on the board and standing for long periods. Low investment in working conditions [51] coupled with the required commute between rural villages and larger urban areas [2] worsen symptoms in the lower limbs of these professionals.

At the severe discomfort level (75 > θ_j_ ≥ 70), daily symptoms were reported in all studied body parts, with emphasis on the thighs, knees, hips, and left foot. Symptoms of daily pain in these regions appear due to high exposure to workloads. Additionally, if working hours were to be reduced, the recovery time of musculoskeletal and joint tissues would be more appropriate. Thus, the effects of excessive workloads can accumulate in the musculoskeletal system and accelerate the development of disorders of the lower limbs [57,58,59,72,74]. In addition, imposed physical demands, prolonged stays at school over several hours, uncomfortable positions, long hours of standing during classes, physical conditions, age, obesity, long sitting times, and frequently climbing of stairs are associated with pain in these regions by education professionals. Therefore, the solution to the development of MSDs in the lower limbs of education professionals involves a broad approach considering all of these factors.

### 4.3. Industrial Workers

The Brazilian footwear industry is characterized by many manual and repetitive activities [75], which justifies the low number of studies that focus on analyzing the symptoms in the lower limbs of workers. However, symptoms in the lower limbs are prevalent in the footwear industry, as this industrial activity has a high number of risk factors of different natures [76,77]. Therefore, MSD has contributed considerably to workers having to resort to the use of muscle relaxants, anti-inflammatories, and analgesics to continue working [78].

Regarding the scale of discomfort in the lower limbs, symptoms were anchored at seven levels, ranging from levels of minimal discomfort (50 > θ_j_ ≥ 45) to severe discomfort (75 > θ_j_ ≥ 70). Thus, the greater number of symptoms anchored at different levels of discomfort strongly indicate that musculoskeletal symptoms in the lower limbs have a greater effect on workers in the footwear industry compared to health and education professionals.

At the minimum discomfort scale level (50 > θ_j_ ≥ 45), workers began to report infrequent symptoms in both thighs. Silva et al. [14] found that symptoms in the thighs of workers in the footwear industry are related to poor postures adopted by workers to alleviate symptoms in body regions that already have symptoms, such as the shoulders and wrists. Miranda Bispo et al. [79] observed that an increase in the weekly frequency of active breaks during the working day to perform workplace exercises could reduce self-reported MSD symptoms. This interruption can reduce tension in several muscles, including the thighs, considering that the workday involves prolonged standing positions [80].

At the mild discomfort level (55 > θ_j_ ≥ 50), infrequent symptoms were also reported in the knees, feet, and hips. According to Leite et al. [77], a worker’s length of service is a risk factor for pain in the lower limbs, with an increased risk of symptoms in the knees, hips, and feet. Therefore, decreased changes in posture can lead to symptoms in other regions of the lower limbs. In addition, jobs requiring cutting and preparation activities may demonstrate symptom prevalence rates in the lower limbs similar to those in the back [81]. The organization of work in the footwear industry still follows Fordist and Taylorist dogmas, which helps explain the high incidence of MSD in these workers [78].

At a low level of discomfort (60 > θ_j_ ≥ 55), workers began to report frequent symptoms in the feet and infrequent pain in the other regions of the lower limbs. Among the three occupational groups, only industrial workers had symptoms in all regions of the lower limbs at this level. Thus, health and education workers experienced the same level of discomfort but reported fewer symptoms than industrial workers. According to Leite et al. [77], bad postures in the footwear industry increase the risk of knee symptoms by over twofold. However, psychosocial stress is also associated with knee symptoms [76]. According to Govindu and Babski-Reeves [82], psychosocial stressors cannot be neglected, as these can affect postures, movements, and application of effort, all of which can affect MSD symptoms.

At the level of moderate discomfort (65 > θ_j_ ≥ 60), frequent symptoms occurred in all regions, except for the hip. At this level, frequent symptoms became a more intense impediment, as these workers use their bodies excessively when working. Kanniappan and Palani [83] highlighted that symptoms in regions such as the knees can prevent everyday activities. In addition, the findings by Silva et al. [18] show that regardless of sex, workers’ hips tend to develop more severe symptoms when symptoms in other regions of the lower limbs are already prevalent. These data are consistent with the findings of our research. Notably, Vieira et al. [84] highlighted that a reduction in stress and increase in physical activity could reduce MSD symptoms in footwear industry workers.

At high discomfort levels (70 > θ_j_ ≥ 65), frequent symptoms were reported in the hip, and daily symptoms were reported in the thighs and feet. Silva et al. [14] found that daily symptoms in the feet of workers in the footwear industry tended to occur earlier than in regions such as the knees. According to Guimarães et al. [85], the Brazilian footwear industry does not provide adequate recovery time for the work demands, which contributes to symptoms in the foot and ankle regions. Another serious factor affecting pain in these regions is employers’ low technological investment in automation [77], making MSD symptoms in the footwear industry a chronic problem.

At the severe discomfort level (75 > θ_j_ ≥ 70), all regions of the lower limbs began to present daily symptoms, with emphasis on the thighs, lower legs, and hips. In this scenario, workers cannot perform their jobs efficiently and take medication to reduce pain, which results in presenteeism in several Brazilian shoe factories [78]. Notably, standing up for an entire working day is common in the footwear industry. This situation is further aggravated by the need to wear safety boots, which are not considered comfortable. Anderson et al. [45] related the use of uncomfortable shoes to the occurrence of symptoms in the feet and hips, and Leite et al. [77] found that age, low social support from supervisors, hostile work environments, and low rewards increase the chance of hip symptoms. In addition, environments with moral harassment increase the chances of symptoms in the hip [76]. Furthermore, the origins and interactions between these factors are complex.

## 5. Conclusions

The objective of this study was to develop a lower limb MSD scale. A cumulative IRT model allowed us to verify that a seven-level scale can measure the progression of MSD symptoms in workers from multiple occupational groups. In addition, using the IRT multigroup model, we verified that the symptoms progressed and accumulated in a specific manner in health, education, and industry professionals. Thus, the professionals’ illness profiles showed different response patterns at the different body regions, even if they presented the same perceived discomfort values at the scale level. These findings suggest that musculoskeletal illnesses occur in different ways and are influenced by the distinct characteristics of the professions in each sector. These results may help ergonomics and safety managers and professionals to better understand MSDs and how these affect the lower limbs of educators, health professionals, and industrial operators.

This study has some limitations. The professionals in this study self-reported their symptoms based on their personal perception. As pain is a subjective assessment, other common tests for musculoskeletal disorders, such as muscle testing in areas of localized pain through neurological exams, should be explored in future studies to increase the reliability of our results. The methods used in this study are robust and reproducible. Nonetheless, the research findings are not generalizable to the other professionals.

## Figures and Tables

**Table 1:** Sample characteristics.

Variable	G1 (N = 167)	G2 (N = 159)	G3 (N = 401)
	n (%)	n (%)	n (%)
Sex			
Men	33 (19.76)	37 (23.27)	350 (87.28)
Women	134 (80.24)	122 (76.73)	51 (12.72)
Age			
18–20	2 (2.98)	5 (3.14)	28 (6.98)
21–30	41 (22.79)	41 (25.78)	223 (55.61)
31–40	51 (30.53)	40 (25.15)	98 (24.44)
41–50	54 (32.33)	54 (33.96)	33 (8.23)
>50	19 (11.37)	19 (11.97)	19 (4.74)
BMI			
Underweight	4 (2.39)	6 (3.77)	16 (3.99)
Normal weight	63 (37.72)	98 (61.63)	192 (47.88)
Overweight	65 (38.92)	40 (25.15)	148 (36.91)
Grade I obesity	24 (14.37)	14 (8.80)	33 (8.23)
Grade II obesity	7 (4.21)	1 (0.65)	10 (2.49)
Grade III obesity	4 (2.39)	0 (0.00)	2 (0.50)
Years at company			
1–5	70 (41.92)	54 (33.96)	203 (50.62)
6–10	52 (31.14)	13 (8.17)	103 (25.69)
11–15	12 (7.18)	28 (17.62)	59 (14.71)
16–20	12 (7.18)	29 (18.24)	23 (5.74)
>20	21 (12.58)	35 (22.01)	13 (3.24)

**Table 2:** MSD symptoms.

Body Region	G1 (N = 167)	G2 (N = 159)	G3 (N = 401)
	n (%)	n (%)	n (%)
Left thigh			
Never	130 (77.84)	110 (69.18)	91 (22.69)
Rarely	14 (8.38)	14 (8.81)	168 (41.90)
Often	11 (6.59)	13 (8.18)	83 (20.70)
Daily	12 (7.19)	22 (13.84)	59 (14.71)
Right thigh			
Never	136 (81.44)	111 (69.81)	115 (28.68)
Rarely	9 (5.39)	21 (13.21)	161 (40.15)
Often	12 (7.19)	9 (5.66)	77 (19.20)
Daily	10 (5.99)	18 (11.32)	48 (11.97)
Left knee			
Never	107 (64.07)	85 (53.46)	183 (45.64)
Rarely	26 (15.57)	34 (21.38)	94 (23.44)
Often	12 (7.19)	17 (10.69)	61 (15.21)
Daily	22 (13.17)	23 (14.47)	63 (15.71)
Right knee			
Never	109 (65.27)	84 (52.83)	181 (45.14)
Rarely	27 (16.17)	33 (20.75)	94 (23.44)
Often	15 (8.98)	20 (12.58)	64 (15.96)
Daily	16 (9.58)	22 (13.84)	62 (15.46)
Left leg			
Never	108 (64.67)	89 (55.97)	222 (55.36)
Rarely	25 (14.97)	24 (15.09)	81 (20.20)
Often	14 (8.38)	13 (8.18)	59 (14.71)
Daily	20 (11.98)	33 (20.75)	39 (9.73)
Right leg			
Never	106 (63.47)	85 (53.46)	225 (56.11)
Rarely	24 (14.37)	30 (18.87)	76 (18.95)
Often	19 (11.38)	15 (9.43)	62 (15.46)
Daily	18 (10.78)	29 (18.24)	38 (9.48)
Hips			
Never	112 (67.07)	78 (49.06)	227 (56.61)
Rarely	22 (13.17)	26 (16.35)	72 (17.96)
Often	10 (5.99)	24 (15.09)	48 (11.97)
Daily	23 (13.77)	31 (19.50)	54 (13.47)
Left foot			
Never	117 (70.06)	103 (64.78)	164 (40.90)
Rarely	14 (8.38)	22 (13.84)	97 (24.19)
Often	17 (10.18)	12 (7.55)	63 (15.71)
Daily	19 (11.38)	22 (13.84)	77 (19.20)
Right foot			
Never	113 (67.66)	101 (63.52)	155 (38.65)
Rarely	16 (9.58)	20 (12.58)	101 (25.19)
Often	21 (12.57)	12 (7.55)	71 (17.71)
Daily	17 (10.18)	26 (16.35)	74 (18.45)

**Table 3:** Factor analysis and estimates of IRT parameters.

Body Region	Parameters					
	F	h2	a	b1	b2	b3
**Group 1**						
Left thigh	0.683	0.467	1.593	1.169	1.696	2.311
Right thigh	0.773	0.597	2.072	1.245	1.565	2.176
Left knee	0.741	0.548	1.875	0.511	1.177	1.593
Right knee	0.741	0.548	1.875	0.539	1.265	1.886
Left leg	0.751	0.563	1.932	0.527	1.170	1.675
Right leg	0.791	0.625	2.197	0.473	1.056	1.679
Hips	0.649	0.422	1.453	0.690	1.379	1.792
Left foot	0.737	0.544	1.858	0.741	1.121	1.750
Right foot	0.708	0.501	1.706	0.674	1.104	1.908
**Group 2**						
Left thigh	0.770	0.593	2.055	0.711	1.096	1.527
Right thigh	0.765	0.586	2.023	0.749	1.340	1.678
Left knee	0.668	0.446	1.527	0.093	0.994	1.625
Right knee	0.726	0.527	1.798	0.123	0.950	1.592
Left leg	0.764	0.584	2.014	0.200	0.792	1.172
Right leg	0.778	0.605	2.108	0.119	0.842	1.261
Hips	0.538	0.289	1.086	−0.049	0.741	1.625
Left foot	0.730	0.533	1.817	0.549	1.148	1.581
Right foot	0.776	0.602	2.091	0.491	0.995	1.363
**Group 3**						
Left thigh	0.680	0.462	1.577	−1.038	0.627	1.599
Right thigh	0.730	0.534	1.820	−0.727	0.731	1.678
Left knee	0.857	0.735	2.835	−0.112	0.568	1.202
Right knee	0.845	0.714	2.691	−0.129	0.575	1.245
Left leg	0.819	0.671	2.433	0.161	0.850	1.660
Right leg	0.822	0.675	2.453	0.190	0.831	1.668
Hips	0.636	0.404	1.402	0.263	1.036	1.768
Left foot	0.880	0.775	3.161	−0.250	0.438	1.003
Right foot	0.862	0.743	2.896	−0.323	0.408	1.065

Note: F, h2, a, b1, b2, and b3 represent the item’s factor loading, commonality, and discrimination values, the difficulty of answer alternative 2, difficulty of answer alternative 3, and difficulty of answer alternative 4, respectively.

**Table 4:** Lower limb discomfort scale.

	Minimal	Mild	Low	Moderate	High	Severe	Maximum
Body Regions	45	50	55	60	65	70	75
**Group 1**							
Left thigh					A2	A3	A4
Right thigh					A2	A3	A4
Left knee				A2	A3	A4	
Right knee				A2	A3	A4	
Left leg				A2	A3	A4	
Right leg			A2		A3	A4	
Hips				A2	A3	A4	
Left foot				A2	A3	A4	
Right foot				A2	A3	A4	
**Group 2**							
Left thigh				A2	A3	A4	
Right thigh				A2	A3	A4	
Left knee			A2	A3		A4	
Right knee			A2	A3		A4	
Left leg			A2	A3	A4		
Right leg			A2	A3	A4		
Hips		A2		A3		A4	
Left foot				A2	A3	A4	
Right foot			A2	A3	A4		
**Group 3**							
Left thigh	A2			A3		A4	
Right thigh	A2			A3		A4	
Left knee		A2		A3	A4		
Right knee		A2		A3	A4		
Left leg			A2	A3		A4	
Right leg			A2	A3		A4	
Hips		A2			A3	A4	
Left foot		A2	A3		A4		
Right foot		A2	A3		A4		

Note: A2 corresponds to an item response indicating that the region “rarely feels pain”, A3 indicates it “often feels pain”, and A4 indicates it “feels pain daily”.

## Data Availability

Not applicable.

## Appendix A. Data Collection Instrument

1–What is your sex: ( ) Male ( ) Female 2–How old are you? _________________ years. 3–What is your weight? _________________ Kg. 4–What is your height? _________________ centimeters. 5–How long have you been in this profession? __________ months 6–What is your professional group? ( ) Health workers ( ) Education workers ( ) Footwear industry workers 7–Indicate the frequency with which you experience some musculoskeletal pain in the following regions of the lower limbs:					
**Diagram**	**Regions**	**Frequency of Pain Symptoms**			
		**Never**	**Rarely**	**Often**	**Daily**
	1–Hips	A	B	C	D
	2–Left thigh	A	B	C	D
	3–Right thigh	A	B	C	D
	4–Left knee	A	B	C	D
	5–Right knee	A	B	C	D
	6–Left leg	A	B	C	D
	7–Right leg	A	B	C	D
	8–Left foot	A	B	C	D
	9–Right foot	A	B	C	D

## Appendix B. Comparison of Response Patterns at Different Levels of the Lower Limb Discomfort Scale

	**Level 45**	**Level 50**	**Level 55**	**Level 60**	**Level 65**	**Level 70**	**Level 75**
**Factors**	**X2** **(*p*-Value)**	**X2** **(*p*-Value)**	**X2** **(*p*-Value)**	**X2** **(*p*-Value)**	**X2** **(*p*-Value)**	**X2** **(*p*-Value)**	**X2** **(*p*-Value)**
**Sex**							
**Category 1 x**							
Category 2	55.69 (0.7008)	3.93 (1.0000)	5.03 (1.0000)	3.51 (1.0000)	5.04 (0.9999)	0.07 (0.9644)	5.25 (0.5125)
Category 3	69.75 (0.9241)	10.31 (1.0000)	14.71 (0.9999)	31.34 (0.7692)	13.74 (0.9995)	2.97 (0.9360)	2.71 (0.6082)
Category 4	65.00 (0.5464)	4.09 (1.0000)	6.92 (0.9999)	10.69 (0.9861)	4.90 (0.9990)	1.50 (0.8262)	4.71 (0.4521)
Category 5	**78.57 (0.0452)**	24.48 (0.5487)	35.06 (0.3247)	27.11 (0.5124)	16.11 (0.8106)	3.88 (0.6923)	6.67 (0.3523)
Category 6	**214.73 (0.0030)**	71.97 (0.8772)	**138.97 (0.0041)**	**90.36 (0.0054)**	41.47 (0.5807)	7.49 (0.8749)	17.03 (0.2545)
**Category 2 x**							
Category 3	36.13 (0.9141)	7.66 (1.0000)	7.07 (0.9826)	11.43 (0.9538)	6.23 (1.0000)	0.57 (0.9893)	0.22 (0.6384)
Category 4	29.03 (0.4109)	2.62 (0.9948)	2.06 (0.9565)	3.80 (0.7040)	1.96 (0.9822)	0.03 (0.8667)	4.83 (0.0894)
Category 5	26.86 (0.1393)	16.74 (0.3344)	11.67 (0.6331)	9.01 (0.6206)	8.64 (0.7331)	0.38 (0.9440)	**8.79 (0.0322)**
Category 6	148.26 (0.0531 *)	49.25 (0.9926)	64.14 (0.9022)	37.70 (0.6599)	23.44 (0.9132)	2.73 (0.9870)	**28.21 (0.0030)**
**Category 3 x**							
Category 4	40.70 (0.9094)	4.00 (1.0000)	5.80 (0.9984)	8.25 (0.9988)	4.28 (1.0000)	0.61 (0.9989)	-
Category 5	43.11 (0.5939)	14.70 (0.9913)	17.14 (0.9049)	8.85 (0.9999)	7.01 (0.9999)	1.23 (0.9987)	-
Category 6	172.42 (0.0823 *)	53.48 (0.9994)	83.94 (0.7133)	29.94 (0.996)	18.72 (1.0000)	3.57 (0.9995)	8.09 (0.5250)
**Category 4 x**							
Category 5	26.63 (0.3747)	6.57 (0.8328)	10.96 (0.8119)	4.48 (0.9918)	3.57 (0.9372)	0.46 (0.9936)	-
Category 6	148.54 (0.0929 *)	31.54 (1.0000)	65.27 (0.9122)	28.29 (0.9757)	14.11 (0.9960)	2.74 (0.9971)	9.09 (0.5233)
**Category 5 x**							
Category 6	129.76 (0.2356)	24.49 (1.0000)	40.06 (1.0000)	25.94 (0.9981)	13.15 (0.9997)	3.16 (0.9987)	10.10 (0.5219)
**Age**							
**Category 1 x**							
Category 2	47.59 (0.6841)	3.29 (1.0000)	4.39 (0.9999)	3.57 (0.9999)	3.48 (0.9990)	0.01 (0.9620)	1.89 (0.7550)
Category 3	21.00 (0.4589)	1.21 (0.9966)	1.24 (0.9900)	0.58 (0.9891)	1.80 (0.9375)	0.02 (0.8786)	4.67 (0.3224)
Category 4	23.45 (0.7101)	0.93 (0.9996)	5.57 (0.9603)	10.34 (0.2420)	4.89 (0.1799)	1.14 (0.5645)	0.89 (0.6386)
Category 5	41.21 (0.4616)	4.32 (1.0000)	2.66 (0.9989)	10.89 (0.9275)	3.41 (0.9991)	0.91 (0.8239)	4.50 (0.2124)
Category 6	18.36 (0.6846)	1.44 (0.9841)	3.23 (0.7793)	4.37 (0.7368)	2.21 (0.8185)	1.08 (0.7827)	0.90 (0.6386)
Category 7	84.92 (0.1415)	29.73 (0.9979)	49.15 (0.9147)	28.90 (0.5226)	15.03 (0.7748)	4.59 (0.9171)	**14.08 (0.04977)**
Category 8	**86.67 (0.0376)**	21.06 (0.8570)	35.44 (0.3540)	**36.08 (0.0298)**	14.59 (0.2645)	1.97 (0.5792)	**16.963 (0.0305)**
Category 9	**38.21 (0.0056)**	2.70 (0.9517)	1.18 (0.9469)	11.39 (0.1806)	3.71 (0.4461)	1.17 (0.5575)	4.50 (0.2124)
**Category 2 x**							
Category 3	42.67 (0.7263)	3.30 (1.0000)	4.40 (0.9995)	2.49 (1.0000)	4.59 (0.9999)	0.08 (0.7692)	3.51 (0.3192)
Category 4	45.06 (0.8522)	3.62 (1.0000)	10.14 (0.9939)	15.70 (0.6769)	7.14 (0.9816)	1.56 (0.4585)	0.56 (0.4542)
Category 5	62.68 (0.6910)	10.50 (1.0000)	5.89 (0.9999)	23.91 (0.7761)	7.55 (1.0000)	1.28 (0.7330)	2.33 (0.3116)
Category 6	39.96 (0.8443)	4.55 (0.9997)	7.72 (0.9723)	8.69 (0.9666)	5.29 (0.9991)	1.84 (0.6054)	0.56 (0.4542)
Category 7	**125.14 (0.0452)**	66.91 (0.4802)	**101.24 (0.0240)**	54.34 (0.079) *	28.04 (0.7541)	6.08 (0.8087)	7.07 (0.3146)
Category 8	**128.34 (0.0089)**	48.55 (0.1950)	**74.10 (0.0030)**	**63.54 (0.0011)**	20.16 (0.7837)	2.99 (0.3929)	8.59 (0.2836)
Category 9	50.76 (0.3278)	7.642 (0.9940)	4.65 (0.9972)	17.15 (0.5798)	7.37 (0.9866)	2.02 (0.3649)	2.33 (0.3116)
**Category 3 x**							
Category 4	18.50 (0.7782)	1.32 (0.9703)	4.62 (0.9482)	4.38 (0.6261)	6.01 (0.6463)	0.63 (0.7302	3.40 (0.0652) *
Category 5	34.73 (0.5760)	4.68 (1.0000)	2.25 (0.9974)	7.56 (0.9750)	5.74 (0.9992)	0.41 (0.9370)	**6.50 (0.0388)**
Category 6	13.40 (0.7676)	1.79 (0.7741)	3.85 (0.4270)	4.51 (0.4784)	4.00 (0.9471)	0.42 (0.9367)	3.40 (0.0652) *
Category 7	74.39 (0.2780)	24.38 (0.9996)	44.78 (0.9513)	17.37 (0.9409)	22.94 (0.5813)	3.14 (0.9778)	**13.833 (0.0316)**
Category 8	75.65 (0.0982) *	17.18 (0.9033)	30.47 (0.4929)	14.96 (0.7788)	19.00 (0.3283)	0.89 (0.8270)	**16.36 (0.0221)**
Category 9	23.852 (0.0676) *	3.03 (0.6952)	1.551 (0.6706)	5.93 (0.4305)	6.11 (0.7285)	0.31 (0.8566)	**6.50 (0.0388)**
**Category 4 x**							
Category 5	35.76 (0.8071)	3.25 (1.0000)	3.58 (0.9998)	5.64 (0.9993)	3.78 (0.9996)	0.45 (0.9783)	-
Category 6	15.68 (0.9241)	1.05 (0.9586)	3.28 (0.9738)	4.14 (0.8439)	2.39 (0.9352)	0.54 (0.9697)	-
Category 7	78.26 (0.3757)	23.71 (0.9998)	51.67 (0.9296)	15.82 (0.9891)	10.38 (0.9825)	3.28 (0.9863)	4.00 (0.4060)
Category 8	79.91 (0.1530)	16.29 (0.9472)	33.12 (0.6517)	14.24 (0.9197)	9.44 (0.8018)	0.96 (0.9160)	5.00 (0.4159)
Category 9	21.65 (0.4807)	1.60 (0.9526)	1.85 (0.9937)	5.21 (0.8156)	2.20 (0.9007)	0.41 (0.9387)	-
Category 5 x							
Category 6	30.237 (0.8110)	3.52 (1.0000)	3.72 (0.9592)	6.77 (0.9953)	2.31 (1.0000)	0.16 (0.9995)	-
Category 7	97.849 (0.2218)	42.31 (0.9964)	61.55 (0.6963)	15.52 (0.9999)	13.26 (0.9995)	3.04 (0.9953)	5.00 (0.4159)
Category 8	100.01 (0.0746) *	29.38 (0.9556)	41.78 (0.2709)	13.14 (0.9995)	9.40 (0.9988)	0.75 (0.9799)	6.00 (0.4232)
Category 9	37.19 (0.3683)	3.98 (1.0000)	1.59 (0.9964)	6.90 (0.9970)	2.82 (1.0000)	0.25 (0.9927)	-
**Category 6 x**							
Category 7	68.32 (0.5005)	18.46 (1.0000)	28.06 (0.9999)	14.965 (0.9899)	8.48 (0.9985)	2.15 (0.9981)	4.00 (0.4060)
Category 8	69.42 (0.2417)	12.22 (0.9847)	13.95 (0.9945)	11.99 (0.9576)	6.54 (0.9811)	0.41 (0.9951)	5.00 (0.4159)
Category 9	15.53 (0.4859)	1.32 (0.8571)	0.49 (0.7843)	5.57 (0.6953)	1.13 (0.9973)	0.07 (0.9994)	-
**Category 7 x**							
Category 8	116.82 (0.3587)	24.78 (1.0000)	40.16 (1.0000)	24.16 (0.9953)	13.47 (0.9974)	3.08 (0.9950)	9.09 (0.5233)
Category 9	70.66 (0.3248)	14.92 (1.0000)	26.48 (0.9999)	15.08 (0.9927)	6.83 (0.9996)	2.71 (0.9940)	5.00 (0.4159)
**Category 8 x**							
Category 9	71.66 (0.1248)	9.42 (0.9988)	12.39 (0.9970)	10.67 (0.9863)	5.88 (0.9816)	0.48 (0.9750)	6.00 (0.4232)
**Length of service**							
**Category 1 x**							
Category 2	50.68 (0.7096)	3.27 (1.0000)	4.30 (0.9998)	3.54 (0.9989)	3.80 (0.9998)	0.07 (0.9644)	0.93 (0.9198)
Category 3	39.49 (0.6652)	3.66 (1.0000)	4.72 (0.9999)	2.92 (0.9999)	4.45 (0.9995)	0.07 (0.9644)	5.41 (0.3682)
Category 4	54.10 (0.8306)	6.00 (1.0000)	10.62 (0.9980)	21.68 (0.4789)	9.16 (0.9954)	1.89 (0.8636)	4.67 (0.3224)
Category 5	51.51 (0.5710)	7.57 (1.0000)	5.46 (0.9997)	16.95 (0.7662)	7.85 (0.9996)	1.91 (0.8618)	
Category 6	42.04 (0.8604)	8.05 (0.9997)	8.54 (0.9973)	8.72 (0.9485)	4.11 (0.9999)	1.73 (0.7849)	0.93 (0.8177)
Category 7	**144.93 (0.0467)**	70.61 (0.7644)	105.50 (0.1263)	**64.13 (0.0489)**	33.42 (0.7217)	8.12 (0.8360)	**23.36 (0.0248)**
Category 8	**115.27 (0.0025)**	36.91 (0.1798)	**57.53 (0.0071)**	**50.54 (0.0008)**	15.91 (0.8913)	2.84 (0.8280)	11.41 (0.0764) *
Category 9	47.96 (0.2786)	14.52 (0.8463)	3.96 (0.9995)	15.15 (0.5846)	8.22 (0.9751)	0.07 (0.9644)	0.93 (0.8177)
**Category 2 x**							
Category 3	21.10 (0.5144)	0.84 (0.9970)	0.75 (0.9798)	0.66 (0.9986)	1.66 (0.8940)	-	2.33 (0.3116)
Category 4	35.72 (0.7766)	1.47 (0.9997)	3.63 (0.9796)	10.61 (0.5623)	4.42 (0.9266)	0.35 (0.8389)	2.12 (0.1449)
Category 5	31.95 (0.4691)	1.78 (1.0000)	1.29 (0.9359)	8.39 (0.7536)	3.61 (0.9895)	0.04 (0.9800)	-
Category 6	23.66 (0.8241)	2.27 (0.9973)	4.06 (0.7733)	4.55 (0.7142)	1.15 (0.9920)	0.03 (0.8663)	-
Category 7	109.28 (16.73)	28.57 (1.0000)	47.64 (0.9926)	31.39 (0.7290)	20.02 (0.7905)	0.02 (0.8663)	11.61 (0.2359)
Category 8	**80.93 (0.0103)**	12.73 (0.6234)	22.57 (0.2076)	**24.67 (0.0256)**	9.49 (0.5766)	2.88 (0.9841)	6.18 (0.1033)
Category 9	29.45 (0.1037)	4.92 (0.5535)	0.22 (0.6384)	7.29 (0.3991)	5.56 (0.3515)	0.04 (0.9979)	-
**Category 3 x**							
Category 4	24.53 (0.7475)	2.77 (0.9935)	4.91 (0.9769)	11.47 (0.6490)	6.05 (0.8104)	-	2.00 (0.3679)
Category 5	19.10 (0.4502)	3.32 (0.9983)	1.72 (0.9735)	10.11 (0.7534)	5.51 (0.9389)	0.35 (0.8389)	1.00 (0.3173)
Category 6	12.44 (0.8236)	3.80 (0.9753)	4.99 (0.8344)	5.46 (0.7928)	2.08 (0.9553)	0.04 (0.9800)	1.00 (0.3173)
Category 7	86.66 (0.3701)	32.10 (0.9998)	59.03 (0.9249)	34.46 (0.6769)	22.61 (0.6520)	0.03 (0.8663)	8.17 (0.6126)
Category 8	**58.14 (0.0400)**	15.73 (0.4000)	**31.96 (0.0437)**	**26.04 (0.0376)**	11.68 (0.3883)	2.88 (0.9841)	4.00 (0.4060)
Category 9	**16.20 (0.0396)**	7.22 (0.3009)	0.52 (0.9146)	8.19 (0.5154)	6.08 (0.2985)	0.04 (0.9979)	1.00 (0.3173)
**Category 4 x**							
Category 5	33.84 (0.7430)	2.43 (1.0000)	3.14 (0.9974)	6.14 (0.9976)	3.89 (0.9996)	-	-
Category 6	26.98 (0.9272)	2.68 (0.9998)	4.28 (0.9967)	5.46 (0.9784)	2.52 (0.9981)	0.50 (0.9920)	-
Category 7	115.03 (0.2162)	31.91 (1.0000)	58.61 (0.9763)	21.86 (0.9979)	13.03 (0.9981)	0.53 (0.9701)	8.09 (0.5250)
Category 8	**85.18 (0.0271)**	14.50 (0.7538)	25.98 (0.4644)	13.39 (0.8602)	4.76 (0.9968)	4.05 (0.9906)	-
Category 9	28.72 (0.4797)	5.24 (0.8747)	1.77 (0.9946)	4.81 (0.9883)	2.31 (0.9933)	0.67 (0.9950)	-
Category 5 x							
Category 6	19.96 (0.8659)	2.81 (1.0000)	3.84 (0.9213)	4.86 (0.9876)	1.97 (0.9999)	0.35 (0.8289)	-
Category 7	94.69 (0.4317)	36.44 (0.9998)	53.18 (0.9784)	19.13 (0.9996)	16.55 (0.9924)	0.08 (0.9993)	7.09 (0.5271)
Category 8	65.36 (0.0851) *	16.82 (0.7732)	26.25 (0.1576)	9.73 (0.9728)	6.59 (0.9932)	2.85 (0.9984)	-
Category 9	19.10 (0.3856)	5.81 (0.9527)	0.66 (0.8819)	4.17 (0.9943)	3.36 (0.9925)	0.11 (1.0000)	-
**Category 6 x**							
Category 7	91.06 (0.5080)	30.17 (1.0000)	48.04 (0.9970)	19.55 (0.9961)	12.47 (0.9950)	0.04 (0.9800)	7.09 (0.5271)
Category 8	61.30 (0.1313)	13.27 (0.8243)	19.64 (0.6052)	10.25 (0.8038)	3.84 (0.9927)	2.85 (0.9965)	-
Category 9	13.84 (0.6783)	4.76 (0.9066)	2.38 (0.7943)	3.66 (0.9223)	1.49 (0.9826)	0.07 (0.9999)	-
**Category 7 x**							
Category 8	123.24 (0.2828)	24.40 (1.0000)	40.06 (1.0000)	26.28 (0.9884)	13.26 (0.9986)	0.03 (0.8663)	10.09 (0.5219)
Category 9	82.62 (0.4600)	19.46 (1.0000)	32.72 (1.0000)	17.52 (0.9988)	10.50 (0.9969)	3.16 (0.9987)	7.09 (0.5271)
**Category 8 x**							
Category 9	53.80 (0.0712) *	5.43 (0.9788)	6.06 (0.9874)	7.81 (0.9310)	2.58 (0.9952)	2.88 (0.9841)	-
**BMI**							
**Category 1 x**							
Category 2	45.59 (0.6878)	3.64 (1.0000)	4.12 (0.9997)	2.23 (0.9975)	4.00 (0.9998)	0.01 (0.9620)	0.90 (0.8256)
Category 3	34.21 (0.5541)	2.16 (0.9996)	1.68 (0.9982)	1.81 (1.0000)	3.34 (0.9992)	0.07 (0.9644)	3.49 (0.6254)
Category 4	47.84 (0.7728)	6.72 (0.9999)	7.81 (0.9885)	16.29 (0.7532)	11.04 (0.9999)	2.41 (0.6611)	2.39 (0.4961)
Category 5	39.06 (0.6008)	3.97 (0.9916)	3.55 (0.9814)	9.24 (0.6819)	2.43 (0.9993)	1.39 (0.8461)	0.11 (0.9481)
Category 6	28.38 (0.6503)	4.86 (0.9932)	0.83 (0.9749)	1.73 (0.9734)	2.43 (0.9984)	1.88 (0.5985)	0.11 (0.9481)
Category 7	**106.92 (0.0074)**	40.38 (0.4981)	43.51 (0.1820)	30.75 (0.0779) *	14.48 (0.9124)	3.06 (0.5485)	6.67 (0.3523)
Category 8	98.42 (0.1188)	35.47 (0.9516)	43.83 (0.9299)	37.02 (0.2888)	31.99 (0.4669)	6.66 (0.7575)	8.60 (0.2823)
Category 9	**70.75 (0.0010)**	12.27 (0.4245)	16.92 (0.0762) *	13.55 (0.3305)	7.57 (0.9749)	3.55 (0.4696)	6.65 (0.2483)
**Category 2 x**							
Category 3	31.47 (0.6837)	2.04 (1.0000)	3.79 (0.9996)	2.87 (0.9993)	2.69 (0.9520)	-	5.62 (0.1315)
Category 4	45.06 (0.8522)	6.65 (1.0000)	10.18 (0.9977)	17.49 (0.6812)	10.04 (0.9988)	0.11 (0.9471)	4.86 (0.027
Category 5	34.85 (0.7749)	3.66 (0.9994)	4.77 (0.9992)	10.26 (0.5933)	1.71 (0.9439)	0.16 (0.9231)	-
Category 6	25.61 (0.7807)	4.68 (0.9993)	2.85 (0.9965)	3.04 (0.8813)	1.59 (0.9025)	0.03 (0.8663)	-
Category 7	**100.32 (0.0226)**	46.51 (0.3694)	**66.38 (0.0126)**	**33.41 (0.0418)**	13.06 (0.6686)	0.91 (0.6333)	**12.60 (0.0133)**
Category 8	92.66 (0.2196)	40.66 (0.9103)	71.95 (0.2876)	40.42 (0.1754)	28.43 (0.2886)	1.46 (0.9933)	**16.07 (0.0067)**
Category 9	**57.69 (0.0212)**	12.84 (0.6149)	24.45 (0.1077)	15.92 (0.1948)	7.65 (0.6628)	0.44 (0.8030)	**13.12 (0.0043)**
**Category 3 x**							
Category 4	33.72 (0.7831)	3.82 (1.0000)	9.21 (0.9547)	19.37 (0.7320)	5.94 (0.9999)	0.70 (0.8737)	6.20 (0.1024)
Category 5	22.84 (0.6934)	1.80 (0.9701)	3.43 (0.9694)	11.38 (0.7253)	0.46 (0.9281)	0.34 (0.9524)	3.58 (0.1667)
Category 6	14.27 (0.6482)	2.58 (0.9787)	0.43 (0.9795)	2.66 (0.9883)	0.54 (0.7619)	0.35 (0.8417)	3.58 (0.1667)
Category 7	**78.18 (0.0480)**	23.86 (0.9228)	**54.96 (0.0171)**	35.87 (0.0565) *	6.20 (0.9388)	1.05 (0.7884)	10.55 (0.1033)
Category 8	70.90 (0.3811)	22.47 (0.9980)	52.99 (0.6614)	45.34 (0.1368)	15.69 (0.8307)	2.11 (0.9895)	12.79 (0.0774) *
Category 9	**45.44 (0.0035)**	7.86 (0.2489)	**23.70 (0.0048)**	15.75 (0.3986)	3.52 (0.8332)	0.89 (0.8270)	**11.30 (0.0457)**
**Category 4 x**							
Category 5	36.61 (0.8627)	2.80 (1.0000)	5.79 (0.9984)	7.17 (0.9988)	4.28 (1.0000)	0.50 (0.9920)	-
Category 6	27.82 (0.8625)	3.48 (1.0000)	4.05 (0.9907)	7.30 (0.9794)	4.16 (1.0000)	0.13 (0.9979)	-
Category 7	**103.36 (0.0344)**	34.08 (0.9354)	46.92 (0.3536)	16.46 (0.9848)	7.81 (1.0000)	0.99 (0.8629)	4.00 (0.4060)
Category 8	96.03 (0.2618)	29.52 (0.9993)	49.41 (0.9472)	17.98 (0.9997)	18.30 (0.9992)	1.53 (0.9996)	5.00 (0.4159)
Category 9	56.49 (0.0814) *	8.10 (0.9856)	16.56 (0.5534)	8.92 (0.9937)	5.42 (1.0000)	0.50 (0.9922)	-
Category 5 x							
Category 6	15.36 (0.8814)	1.73 (0.9950)	1.04 (0.9595)	1.81 (0.9864)	-	0.40 (0.9826)	-
Category 7	73.27 (0.2254)	17.43 (0.9943)	43.95 (0.1702)	10.61 (0.9798)	2.74 (0.9938)	1.21 (0.9439)	3.00 (0.3916)
Category 8	67.00 (0.7052)	17.40 (0.9999)	41.75 (0.9567)	11.18 (0.9999)	8.56 (0.9875)	2.61 (0.9939)	4.00 (0.4060)
Category 9	33.95 (0.2410)	4.92 (0.5539)	17.11 (0.0719) *	3.48 (0.9956)	1.31 (0.9341)	1.07 (0.9564)	-
**Category 6 x**							
Category 7	64.98 (0.1680)	21.13 (0.9831)	20.29 (0.9087)	13.42 (0.7074)	2.32 (0.9933)	0.96 (0.9160)	3.00 (0.3916)
Category 8	58.51 (0.6702)	20.15 (0.9998)	16.40 (1.0000)	13.74 (0.9926)	7.72 (0.9893)	1.45 (0.9991)	4.00 (0.4060)
Category 9	29.50 (0.0585) *	6.15 (0.6300)	3.91 (0.4182)	4.14 (0.8447)	1.03 (0.9055)	0.43 (0.9801)	-
**Category 7 x**							
Category 8	106.52 (0.4676)	22.80 (1.0000)	35.86 (1.0000)	22.09 (0.9966)	11.65 (0.9989)	2.71 (0.9940)	7.08 (0.5271)
Category 9	**80.38 (0.0489)**	11.35 (0.9999)	24.40 (0.9103)	14.57 (0.8799)	3.11 (0.9995)	1.47 (0.9159	6.00 (0.4232)
**Category 8 x**							
Category 9	72.99 (0.3800)	13.74 (1.0000)	20.57 (1.0000)	14.37 (0.9987)	9.94 (0.9948)	1.94 (0.9987)	7.00 (0.4289)
Note 1: For the sex factor, Category 1 (male healthcare professional), Category 2 (female healthcare professional), Category 3 (male healthcare professional), Category 4 (female healthcare professional), Category 5 (male professional in the footwear industry), and Category 6 (female professional in the footwear industry). Note 2: For the age factor, Category 1 (health professional <30 years old), Category 2 (health professional aged 30–49 years), Category 3 (health professional >50 years old), Category 4 (education professional <30 years old), Category 5 (education professional aged 30–49 years), Category 6 (education professional >50 years old), Category 7 (industry professional footwear industry <30 years old), Category 8 (footwear industry professional aged 30–49 years), and Category 9 (footwear industry professional >50 years old). Note 3: For the length of service factor, Category 1 (health professional with <10 months of service time), Category 2 (health professional with service time between 10 and 20 months), Category 3 (health professional with >20 months of service time), Category 4 (education professional with <10 months of service), Category 5 (education professional with service time between 10 and 20 months), Category 6 (education professional with >20 months of service time), Category 7 (footwear industry professional with <10 months of service time), Category 8 (footwear industry professional with service time between 10 and 20 months), and Category 9 (professional from the footwear industry with >20 months of service time). Note 4: For the BMI factor, Category 1 (underweight or normal weight healthcare professional), Category 2 (overweight healthcare professional), Category 3 (obese healthcare professional), Category 4 (underweight or normal weight education professional), Category 5 (overweight education professional), Category 6 (obese education professional), Category 7 (underweight or normal weight footwear industry professional), Category 8 (professional overweight footwear), and Category 9 (obese footwear industry professional). Note 5: Values with *p*-value < 0.05 are in bold; Values with * are close to statistical significance. The character—indicates the absence of individuals in the sample with a given characteristic at a given level of risk on the scale.							
